# A Systematic Review of Palmitate-Mediated Insulin Resistance in C2C12 Myotubes

**DOI:** 10.3390/nu17223619

**Published:** 2025-11-20

**Authors:** John M. Zimmerman, Alexa J. Klein, Kipton B. Travis, Roger A. Vaughan

**Affiliations:** Department of Health and Human Performance, High Point University, High Point, NC 27268, USA; jzimmerm@highpoint.edu (J.M.Z.); aklein4@highpoint.edu (A.J.K.); ktravis@highpoint.edu (K.B.T.)

**Keywords:** C2C12 myotubes, palmitate, insulin resistance, p-Akt, glut4, skeletal muscle

## Abstract

Background/Objectives: Skeletal muscle plays a pivotal role in whole-body glucose metabolism and is a major target in the pathogenesis and treatment of insulin resistance and type 2 diabetes. The C2C12 myotube cell line is one of the most used in vitro models to investigate mechanisms of insulin resistance. This systematic review (1) summarizes the most common experimental conditions including palmitate concentrations and treatment durations used to induce insulin resistance in C2C12 myotubes; (2) characterizes outcomes related to insulin resistance; and (3) discusses strengths and limitations associated with this model. Methods: A systematic search of PubMed and Scopus was conducted using terms “C2C12 AND palmitate AND insulin resistance” and related variations. A total of 191 articles met inclusion criteria. Results: The most frequently used palmitate concentrations were 0.25 mM, 0.5 mM, and 0.75 mM for at least 16 h, which consistently led to decreased insulin-stimulated pAkt expression, GLUT4 abundance, and insulin-stimulated glucose uptake. Conclusions: The high volume and consistency of primary findings is a key strength of this article which demonstrated reduced insulin signaling across various culture conditions, treatment durations, and insulin co-stimulation protocols.

## 1. Introduction

Skeletal muscle is a metabolically important and active contributor to human movement and physiology. Skeletal muscle is also a consequential tissue for several pathologies such as insulin resistance and diabetes, due to its key role in glucose metabolism. For this reason, skeletal muscle is a primary target tissue for improving insulin sensitivity for the benefit of metabolic disease. Thus, exploring the mechanisms of insulin resistance in skeletal muscle is noble and offers the possibility of treatments. Among the most common experimental proof-of-concept strategies is the use of in vitro systems such as the myoblast cell culture line, C2C12. Originally, C2C12 cells were isolated from mouse skeletal muscle and can differentiate into multinucleated myotubes. Moreover, it is known to be insulin-responsive and is recognized as a convenient and effective model for studying several attributes of muscle physiology during health and disease including insulin resistance [1].

While there are other methods of inducing insulin resistance in vitro, treatment of cultured myotubes with the saturated fatty acid, specifically palmitate, is among the most widely used. A prodigious number of primary research articles have utilized this technique for inducing insulin resistance, and many of these manuscripts have focused on methods of restoring the palmitate-mediated reduction in insulin signaling. Given the widespread use of palmitate-treated myotubes as a model of insulin resistance, and because of how common the palmitate-treated C2C12 myotube model has become, there is a need to summarize the common features of this model. Therefore, this systematic review was undertaken to provide a summary of common features of palmitate-mediated insulin resistance in C2C12 myotubes.

### 1.1. Mechanistic Overview of Palmitate-Mediated Insulin Resistance

**Figure 1:**
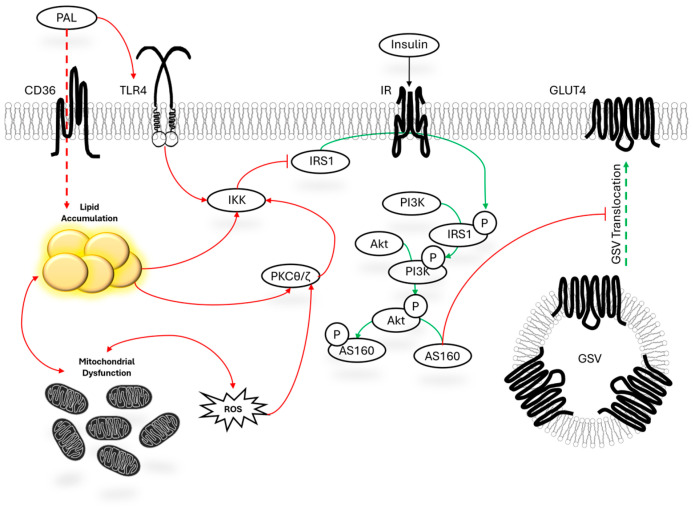
Mechanistic overview of palmitate-induced insulin resistance. Notes: Solid black arrows denote signaling and activation. Arrows that travel behind a molecular target indicate that the molecular target over the arrow phosphorylates the target from which arrow originates. Green arrows indicate normal insulin signaling mechanisms. Red arrows indicate the contribution of this pathway negatively impacts insulin signaling. Dashed arrows indicate the uptake of palmitate or translocation of GLUT4 vesicles. Abbreviations: GLUT4, glucose transporter member 4; GSV, GLUT4 storage vesicles; Iκκ, I kappa B kinase; IRS1, insulin receptor substrate 1; IR, insulin receptor; PI3K, phosphatidylinositol 3-kinase; PKC, protein kinase c; ROS, reactive oxygen species; TLR4, toll-like receptor 4.

In general, several mechanisms have been proposed for how palmitate promotes insulin resistance, much of which has been described elsewhere [2,3]. Briefly, the accumulation of various lipid subtypes in skeletal muscle may disrupt several signaling cascades; observations which date back to 1999 [4]. For example, increased lipid accumulation including ceramides, diacylglycerols, and other lipids can activate inflammatory signaling such as I kappa B kinase (Iκκ) (directly by lipid excess or via increased protein kinase c (PKC)). PKC and Iκκ activation can ultimately inhibit insulin receptor substrate 1 (IRS1), thereby reducing cell responsiveness to insulin stimulation. Reduced IRS1 response to insulin stimulation results in reductions in Akt activation, glucose transporter 4 (GLUT4) abundance, and GLUT4 translocation to the cell membrane. Additionally, lipid toxicity is also associated with increased reactive oxygen species production, which is associated with organelle dysfunction, especially mitochondrial dysfunction. Collectively, reduced response within the canonical insulin signaling pathway, reduced mitochondrial function, and heightened reactive oxygen species (ROS) are all common features of palmitate-mediated insulin resistance in C2C12 myotubes (Figure 1). Detailed mechanistic overviews of lipid mediated insulin resistance are available at the following reviews [2,3].

### 1.2. Aims and Scope of Review

Given the increasing prevalence of insulin resistance and diabetes, experiments that assess basic attributes and mechanisms for potential therapeutics are important. Because palmitate-mediated insulin resistance in C2C12 myotubes is a commonly used model for assessing insulin resistance, this work aims to (1) highlight the concentrations and durations commonly used in palmitate-mediated insulin resistance in C2C12 myotubes; (2) describe the effects of palmitate-mediated insulin resistance in C2C12 myotubes on various outcomes associated with both basal and insulin signaling response; and (3) discuss the current obstacles and considerations associated with this model. In general, the most frequently used palmitate concentrations were 0.25 mM, 0.5 mM, and 0.75 mM for at least 16 h, which consistently led to decreased insulin-stimulated pAkt expression, GLUT4 abundance, and insulin-stimulated glucose uptake. Obstacles such as highly variable culture conditions, insulin stimulation protocol, use of far beyond pharmacokinetically attainable levels of palmitate and loss of cell viability are among the most prominent obstacles in the use of palmitate-treated C2C12 myotubes as a model for skeletal muscle insulin resistance. Importantly, multiple other in vitro models and cell lines are also commonly used but not included (such as L6 or Primary Human Skeletal Muscle Cells (HSkMC)). However, C2C12 myotubes were focused on in the present report because it is among the most used model of mimicking skeletal muscle in vitro.

## 2. Materials and Methods

### 2.1. General Article Search Procedure and Eligibility Criteria

Primary literature was identified by RAV first by searching PubMed and Scopus using individual “C2C12 AND palmitate AND insulin resistance”. The search was then repeated with “palmitate” substituted with “palmitic acid” both with the addition of each targeted outcome (for example “pAkt”) both with and without the presence of “insulin resistance”. Initially, full texts for each article were screened by RAV (as the abstract is insufficient in many cases to know if eligible experiments were contained within the manuscript). Articles were included and summarized if (1) C2C12 myotubes were used as the cell culture model of insulin resistance, (2) the article included a palmitate-only group along with appropriate vehicle or true control, (3) the article assessed at least one of the related outcomes associated with the canonical insulin signaling pathway of importance to the scope of the review (per PRISMA guidelines, the outcomes are included as column topics within each table). Additionally, when appropriate, experiments with modified C2C12 cell line (i.e., reporter, siRNA, etc.) were included if relevant comparisons and outcomes were included with corresponding control.

### 2.2. Secondary Article Screening and Inclusion Procedure

All references were initially screened for eligibility by a single reviewer (RAV) with curation completed using the publication date of 19 June 2025 (and final article curation completed as of 8 October 2025). Articles which were initially excluded were then reassessed for eligibility criteria by a second independent author (JMZ), and incorrectly omitted articles were also included and summarized. Articles that were deemed eligible following initial screening were then re-evaluated by a second independent member of the research team (JMZ, AJK, and KBT), at which point measurements/estimates of effects for each outcome were made. Estimates of outcomes were generated using ratio-metric measurements of palmitate treatment versus relevant control (or raw data was reported when available). Importantly, although quality control and risk of bias is essential to guarantee the scientific quality of each individual study, standards of reporting for in vitro experiments have changed substantially since the introduction of the model. Moreover, limited risk-of-bias assessment tools are validated for in vitro studies (specifically related to the presented outcomes), therefore a bias assessment was not performed for each report.

Additionally, because the concentrations of palmitate used in the literature varied widely (from 0.05–1.2 mM), we stratified the results from each included paper into 3 tiers of concentrations. These tiers are (1) those that used pharmacokinetically attainable levels which include concentrations ≤0.25 mM, (2) those that used palmitate concentrations that moderately exceed pharmacokinetically attainable levels ranging from 0.251–0.5 mM, and (3) those which well-exceed pharmacokinetic levels (proof-of-concept), which include treatment conditions >0.5 mM palmitate. The determination of pharmacokinetically attainable concentrations was based on observations showing levels of palmitate approximate 0.007–0.015 mM (fasting [5]) up to 0.145–0.210 mM (post-prandial [6]). Additionally, to provide a summary of the most relevant treatment conditions, a general estimate of the effect of the most common concentration and treatment duration was summarized in text for each outcome as the average relative values of palmitate treatments versus control (control = 100) following variable insulin stimulation conditions (presented as total experimental average ± SD versus relative control). Importantly, these estimated average effects do not include data for protein expression only quantified by visual confirmation (VC). Additionally, within some individual studies, multiple distinct experiments were conducted involving differing treatment conditions (such as varying treatment duration) and are therefore provided as independent rows within each Table.

## 3. Results

### 3.1. Search Results

**Figure 2:**
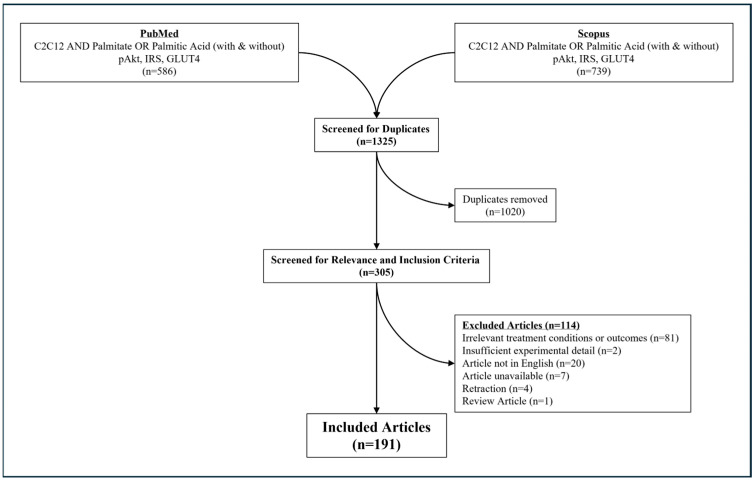
Flowchart of search strategy and results from PubMed and Scopus search. Search strategy for palmitate-mediated insulin resistance in C2C12 myotubes using “C2C12 AND palmitate AND insulin resistance” with and without targets related to insulin resistance as outlined in Methods and figure above.

An initial historical search identified an article published as early as 1999 which first assessed the effect of palmitate conjugated with BSA on insulin signaling in C2C12 cells [4]. Initial search results from both PubMed and Scopus identified 1325 search results. After removing duplicate articles from overlapping search results, a total of 310 articles were identified and twice-screened (initially by RAV and secondarily by a separate independent author for verification of inclusion criteria and generation of estimated effect). After further removal of articles without relevant experimental conditions and/or outcomes, unavailable texts, review articles, articles not in English, and retractions, a total of 191 articles were included (Figure 2). Further details regarding article inclusion/exclusion results (Supplementary Table S1) and raw data estimates (Supplementary File S1) are provided within the Supplemental Materials.

### 3.2. Organization of Data

We examined the effect of palmitate at various concentrations on insulin signaling in C2C12 myotubes, analyzing changes in phosphorylated IRS (pIRS), phosphorylated Akt (pAkt), GLUT4, and glucose uptake under both basal and insulin-stimulated states. As noted above, under normal conditions, insulin binds to its receptor and activates IRS via phosphorylation. Downstream, this activates Akt through phosphorylation, which promotes GLUT4 translocation to the cell membrane to ultimately promote glucose uptake. Therefore, to reveal the effect of palmitate on insulin signaling, we highlight the response of myotubes following insulin stimulation, which reveals the effect on responsiveness to insulin. Though, for reference and completeness, we also include the results from basal experiments within each Table. Also as noted above, because the concentrations of palmitate used in the literature varied widely (from 0.05–1.2 mM), we have stratified the results into 3 tiers of concentrations including those that approximate pharmacokinetically attainable levels which include concentrations ≤0.25 mM (Table 1). These concentrations were chosen based on observations demonstrating levels of palmitate approximate 0.007–0.015 mM (fasting [5]) up to 0.145–0.210 mM (post-prandial [6]). Additionally, other experiments were stratified as those that used palmitate concentrations that moderately exceed pharmacokinetically attainable levels ranging from 0.251–0.5 mM (Table 2), and those which well-exceeded pharmacokinetic (proof-of-concept) levels including treatment conditions >0.5 mM (Table 3). Moreover, to provide a summary of the most relevant treatment conditions, a general estimate of the effect of the most common concentration and treatment duration was summarized in text for each outcome as the average relative values of palmitate treatments versus control (control = 100%) following variable insulin stimulation conditions (presented as average ± SD). Importantly, these estimated average effects do not include data for protein expression only quantified by VC.

### 3.3. Effect of Pharmacokinetically Attainable Levels of Palmitate on Insulin Signaling

We began by assessing the reported effects of pharmacokinetically attainable palmitate levels (concentrations ≤ 0.25 mM), which accounted for about 1/3 of the experimental observations, on common indicators of insulin sensitivity. Expression of pAkt following insulin stimulation was the most common measurement of the inclusion criteria outcomes. Within the experiments that utilized pharmacokinetically attainable levels, 0.2 mM for 24 h was the most common set of treatment conditions. Cells treated with 0.2 mM for 24 h exhibited decreased pIRS expression (total experimental average 37.9 ± 17.0% versus control following variable insulin stimulation). Similar reductions in pAkt expression, GLUT4, and glucose uptake were observed following treatment with 0.2 mM for 24 h (total experimental average for pAkt (43.1 ± 23.2%), GLUT4 (70.0 ± 19.9%), and glucose uptake (56.0 ± 19.3%), following variable insulin stimulation conditions). Additional experimental results for all included articles that utilized treatments with pharmacokinetically attainable levels of palmitate are included in Table 1.

### 3.4. Effect of Moderately Higher than Pharmacokinetically Attainable Levels of Palmitate on Insulin Signaling

Next, we continued summarizing the effect of palmitate on insulin sensitivity by assessing the reported effect of experiments that utilized moderately higher than pharmacokinetically attainable levels (concentrations 0.251–0.5 mM), which, again accounted for about 1/3 of the experimental observations. Consistent with findings from Table 1, expression of pAkt following insulin stimulation was again the most common measurement of the inclusion criteria outcomes. Within the experiments that utilized moderately higher than pharmacokinetically attainable levels, 0.5 mM for 16 h was the most common set of treatment conditions. Following treatment with 0.5 mM for 16 h, the palmitate-treated cells exhibited mixed effects on pIRS expression (total experimental average 136.4 ± 69.0% versus control following variable insulin stimulation). However, like pharmacokinetically attainable levels of palmitate, 0.5 mM palmitate consistently reduced pAkt expression (57.6 ± 21.5%), GLUT4 (48.0 ± 22.2%), and glucose uptake (61.5 ± 23.8%), following variable insulin stimulation conditions. Additional experimental results for all included articles that utilized treatments with moderately higher than pharmacokinetically attainable levels of palmitate are included in Table 2.

### 3.5. Effect of “Proof-of-Concept” Levels of Palmitate on Insulin Signaling

Lastly, we completed summarizing the effect of palmitate on insulin sensitivity by assessing the reported effect of experiments that utilized concentrations of palmitate that are far higher than pharmacokinetically attainable levels or “proof-of-concept” levels (concentrations > 0.5 mM), which accounted for roughly the final 1/3 of the experimental observations. Once again, expression of pAkt following insulin stimulation remained the most observed measurement of the inclusion criteria outcomes. Within the experiments that utilized proof-of-concept levels, 0.75 mM for 16 h was the most common set of treatment conditions. Unlike moderately high levels of palmitate, treatment with 0.75 mM palmitate for 16 h displayed relatively consistent reductions in pIRS expression (total experimental average 68.9 ± 71.8% versus control following variable insulin stimulation). Moreover, treatment with 0.75 mM palmitate for 16 h also consistently reduced pAkt expression (50.1 ± 14.6%), GLUT4 (55.3 ± 39.6%), and glucose uptake (61.0 ± 24.7%), following variable insulin stimulation conditions. Additional experimental results for all included articles that utilized proof-of-concept levels of palmitate treatments are described in Table 3.

## 4. Discussion

The present systematic review examined 191 published studies that investigated palmitate-induced insulin resistance in C2C12 myotubes. In general, the most frequently used palmitate concentrations were 0.25 mM, 0.5 mM, and 0.75 mM for at least 16 h, which consistently led to decreased insulin-stimulated pAkt expression, GLUT4 abundance, and insulin-stimulated glucose uptake. Within this review, we placed an emphasis on the most common treatment conditions for each level of palmitate treatment used within experiments. Collectively, the results from the studies reveal well-conserved effects of palmitate exposure on insulin signaling and glucose uptake.

### 4.1. IRS

IRS activation following insulin stimulation is considered a component of the proximal canonical insulin signaling pathway [197]. The most inconsistent finding of our review was the effect of palmitate treatment on IRS activation following insulin stimulation. Of the most common treatment conditions at pharmacokinetically attainable palmitate levels, pIRS expression was consistently reduced. Conversely, experiments that utilized moderately higher than pharmacokinetically attainable palmitate levels exhibited mixed results. However, proof-of-concept concentrations which far exceed the pharmacokinetically attainable palmitate levels of palmitate found reduced IRS activation following insulin stimulation. Such disparities may be due to a concentration-dependent effect during which, at low levels, palmitate impedes the action of insulin signaling in the mechanism outlined in Figure 1. However, at levels moderately higher than those which are pharmacokinetically attainable, palmitate may activate IRS via mTORC-dependent mechanisms which can further reduce insulin sensitivity [78]. Finally, at proof-of-concept concentrations, palmitate again reduces the activation of IRS possibly due to off-target mechanisms (such as reduced cell viability, discussed below). Thus, additional dose-dependent studies should investigate the importance of timing and concentration of palmitate treatment to elucidate if the disparate findings across concentrations are due to experimental variability, or the speculated hypothesis outlined above involving both concentration-dependent effects confounded by cytotoxicity.

### 4.2. Akt

Unlike IRS, Akt activation following insulin stimulation was the most common measurement and most consistent finding in the present review. In fact, consistently reduced pAkt expression following insulin stimulation was observed across all levels of palmitate treatment. That said, like IRS, Akt activation is considered a component of the proximal canonical insulin signaling pathway. As such, it has been discussed that under some experimental circumstances, large changes in Akt activation may not necessarily equate to large changes in the activation/inactivation of downstream targets, and that loss of Akt activation alone may not explain the entirety of the status of insulin resistance [197]. This ‘spareness’ exhibited by Akt and other proximal signaling molecules implies that only small amounts of function of such signals may be required to elicit the physiological response.

### 4.3. GLUT4

Unlike IRS and Akt activation, which are components of the proximal canonical insulin signaling pathway, GLUT4 vesicle translocation to the plasma membrane does not exhibit the same ‘spareness’ as IRS and Akt activation and is therefore less debated on its importance during insulin resistance. Thus, an important finding within this report is that GLUT4 levels were consistently reduced in the most common treatment conditions from all three categories of palmitate concentrations. However, an important limitation of these measurements was that several reports did not assess membrane translocation. That said, of the reports that assessed both GLUT4 levels and glucose uptake, it appears that when GLUT4 levels were reduced, so were levels of glucose uptake.

### 4.4. Glucose Uptake

The final indicator of insulin sensitivity assessed in the current report is the uptake of glucose, which is not a portion of signaling cascade, but rather the mechanistic outcome of the functional cascade. Like Akt activation and GLUT4 levels, glucose uptake was consistently reduced across all three levels of palmitate treatment. And as noted above, a striking consistency between GLUT4 abundance and glucose uptake was observed across most reports that assessed both outcomes. However, important disparities across reports are worthy of consideration. For example, the duration of fasting (serum, glucose, or both) and composition of media components (high versus low glucose) both before and during the insulin stimulation experiments. Similarly, the duration of and concentration of insulin treatment also varied substantially between reports.

### 4.5. Limitations and Considerations

In general, the collective results from the summarized experiments align with impaired insulin signaling and are consistent with the mechanisms of lipid-induced insulin resistance, including the hypothesis that excess saturated fatty acids disrupt insulin receptor substrate (IRS1) signaling, impair phosphatidylinositol 3-kinase (PI3K)/Akt pathway activity, and reduce downstream glucose uptake [2,3]. However, it is acknowledged that the multifaceted nature of lipid toxicity in skeletal muscle (such as its capacity to promote inflammation, impair other signaling cascades, and disrupt metabolic homeostasis) are other topics worthy of comment. Therefore, a limitation of the current review is the selective scope which included only the effect of palmitate treatment on insulin signaling and glucose uptake. Future comments on the effect of palmitate treatment on markers of inflammation and metabolic dysfunction are warranted.

Additionally, several limitations are worth noting. First, the preparation and conjugation of palmitate to bovine serum albumin (BSA) is a critical step in ensuring bioavailability and reducing toxicity. This step varied across the studies, resulting in a lack of standardization. This methodological variability could influence both the magnitude and reproducibility of the observed effects. Secondly, the duration and concentration of insulin stimulation used to assess insulin responsiveness varied widely, making direct comparisons across independent studies more complex. Thirdly, C2C12 myotubes differ from primary human skeletal muscle (in vitro and in vivo) in several keyways. Moreover, the in vitro C2C12 model lacks interplay with other tissues and can only serve as a simplified model in this context. Thus, while the C2C12 myotubes are useful for insight regarding the mechanisms, findings from this model may not provide full insight into the physiological mechanisms and responses noted in human tissue. And finally, a major limitation worthy of considering is the common use of palmitate levels that far exceed pharmacokinetically attainable levels. This is important as the physiological relevance of these findings for in vivo systems is questionable. Moreover, several reports noted loss of cell viability at several of the utilized concentrations (most commonly >0.3 mM). In fact, several reports noted loss of cell viability (usually assessed via tetrazolium assay) at pharmacokinetically attainable levels [9,13,21,31,42,195], though it was more common for these lower concentrations to not affect viability. However, these observations raise questions about the off-target mechanisms of palmitate concentrations that exceeded these levels.

### 4.6. Strengths and Conclusions

Despite the limitations highlighted above, our review has several strengths. First, a strength of this report is the volume and consistency of the primary findings. The reliability of reduced insulin signaling following palmitate treatment despite differences in culture conditions, treatment durations, and insulin co-stimulation protocols across various studies demonstrates a high degree of reproducibility of the model. Indeed, reductions in insulin-stimulated pAkt and GLUT4 levels as well as consistently reduced insulin-stimulated glucose uptake provide convincing evidence of the reliability in palmitate-treated myotubes as a model of skeletal muscle insulin resistance. These shared features across studies emphasize and reinforce the validity and utility of the palmitate–C2C12 model as a robust in vitro system for probing mechanisms related to insulin resistance.

Collectively, this report demonstrates the highly consistent dose- and time-dependent effects of palmitate-mediated insulin resistance in the C2C12 myotube model of skeletal muscle. The findings herein may also be useful by informing future studies of in vitro models of insulin resistance and serve as a user’s reference for C2C12 myotube insulin resistance.

## Figures and Tables

**Table 1:** Findings from systematic search results on the effect of pharmacokinetically attainable levels of palmitate treatment at various concentrations for various durations on common indicators of insulin sensitivity.

Palmitate Concentration	Duration	Vehicle	Insulin Stimulation	Insulin Receptor Substrate (pIRS)	Protein Kinase B (pAkt)	Glucose Transporter 4 (GLUT4)	Glucose Uptake	Reference
0.05 mM	16 h	BSA	17.2 nM × 15 min		↓ (≈24.6 ± 10%)			De Wilde et al., 2010 [7]
0.0625 mM	24 h	BSA	No		↓VC			Cheon et al., 2014 [8]
0.0625 mM	24 h	BSA	100 nM × 30 min		↓VC			Cheon et al., 2014 [8]
0.075 mM	24 h	BSA	No		↓ (≈39.1 ± 10%)		↓ (≈52.5 ± 10%)	Huang et al., 2024 [9]
0.1 mM	12 h	BSA	Unknown		↔ VC			Chen et al., 2016 [10]
0.1 mM	16 h	BSA	17.2 nM × 15 min		↓ (≈32.0 ± 10%)			De Wilde et al., 2010 [7]
0.1 mM	16 h	BSA	100 nM × 10 min		↓ VC			Chavez et al., 2005 [11]
0.1 mM	16 h	BSA	No				↔ (≈100.0 ± 5%) SEM	Na et al., 2025 [12]
0.1 mM	16 h	BSA	200 nM × 20 min				↓ (≈61.0 ± 5%) SEM	Na et al., 2025 [12]
0.1 mM (3D)	16 h	BSA	No				↔ (≈100.0 ± 5%) SEM	Na et al., 2025 [12]
0.1 mM (3D)	16 h	BSA	200 nM × 20 min				↓ (≈50.0 ± 5%) SEM	Na et al., 2025 [12]
0.1 mM	18 h	BSA	No				↓ (≈83.6 ± 5%)	Park et al., 2014 [13]
0.1 mM	18 h	BSA	100 nM × 10 min				↓ (≈61.4 ± 5%)	Park et al., 2014 [13]
0.1 mM	24 h	BSA	No	↓ (≈65.7 ± 5%)		↓ (≈60.5 ± 5%)	↓ (≈60.0 ± 5%)	Ren et al., 2025 [14]
0.1 mM	24 h	BSA	10 nM × 30 min		↓ (≈85.7 ± 5%)	↓ (≈84.0 ± 5%)		Norouzi et al., 2019 [15]
0.1 mM	24 h	BSA	100 nM × 30 min	↔ (≈108.3 ± 5%)	↔ (≈85.3 ± 5%)	↔ (≈92.5 ± 5%)	↔ (≈109.0 ± 5%)	Pan et al., 2023 [16]
0.1 mM	24 h	BSA	No	↔ (≈72.7 ± 5%)		↔ (≈100.0 ± 5%)		Zhang et al., 2018 [17]
0.1 mM	24 h	BSA	No		↔ (≈95.0 ± 5%)			Hirabara et al., 2010 [18]
0.1 mM	24 h	BSA	7 nM × 30 min		↓ (≈51.0 ± 5%)			Hirabara et al., 2010 [18]
0.1 mM	24 h	BSA	100 nM × 30 min			↔ (≈93.0 ± 10%)	↔ (≈96.0 ± 5%)	Zhang et al., 2015 [19]
0.125 mM	16 h	BSA	No				↔ (≈100.0 ± 5%) SEM	Feng et al., 2012 [20]
0.125 mM	16 h	BSA	100 nM × 30 min				↔ (≈91.6 ± 5%) SEM	Feng et al., 2012 [20]
0.125 mM	24 h	BSA	No	↓ VC			↓ (≈71.8 ± 5%) SEM	Cheon et al., 2014 [8]
0.125 mM	24 h	BSA	100 nM × 30 min	↓ VC			↓ (≈67.0 ± 5%) SEM	Cheon et al., 2014 [8]
0.15 mM	16 h	BSA	No				↔ (≈100.0 ± 5%) SEM	Na et al., 2025 [12]
0.15 mM	16 h	BSA	200 nM × 20 min				↓ (≈61.0 ± 5%) SEM	Na et al., 2025 [12]
0.15 mM (3D)	16 h	BSA	No				↔ (≈100.0 ± 5%) SEM	Na et al., 2025 [12]
0.15 mM (3D)	16 h	BSA	200 nM × 20 min				↓ (≈35.7 ± 5%) SEM	Na et al., 2025 [12]
0.15 mM (LG)	24 h	BSA	No				↔ (≈86.2 ± 5%)	Kopp et al., 2023 [21]
0.15 mM (LG)	24 h	BSA	100 nM × 20 min				↔ (≈84.6 ± 5%)	Kopp et al., 2023 [21]
0.15 mM (HG)	24 h	BSA	No				↓ (≈68.8 ± 5%)	Kopp et al., 2023 [21]
0.15 mM (HG)	24 h	BSA	100 nM × 20 min				↓ (≈82.8 ± 5%)	Kopp et al., 2023 [21]
0.15 mM (LG)	96 h	BSA	No				↔ (≈113.6 ± 5%)	Kopp et al., 2023 [21]
0.15 mM (LG)	96 h	BSA	100 nM × 20 min				↔ (≈87.9 ± 5%)	Kopp et al., 2023 [21]
0.15 mM (HG)	96 h	BSA	No				↓ (≈67.2 ± 5%)	Kopp et al., 2023 [21]
0.15 mM (HG)	96 h	BSA	100 nM × 20 min				↓ (≈77.4 ± 5%)	Kopp et al., 2023 [21]
0.2 mM	8 h	BSA	No		↔ (≈94.4 ± 5%)	↔ (≈30.7 ± 5%)		Mok et al., 2020 [22]
0.2 mM	8 h	BSA	100 nM × 60 min		↓ (≈49.3 ± 5%)	↓ (≈44.8 ± 5%)		Mok et al., 2020 [22]
0.2 mM	12 h	BSA	Unknown		↓ VC			Chen et al., 2016 [10]
0.2 mM	16 h	BSA	No				↔ (≈100.0 ± 5%) SEM	Na et al., 2025 [12]
0.2 mM	16 h	BSA	200 nM × 20 min				↓ (≈55.9 ± 5%) SEM	Na et al., 2025 [12]
0.2 mM (3D)	16 h	BSA	No				↔ (≈118.2 ± 5%) SEM	Na et al., 2025 [12]
0.2 mM (3D)	16 h	BSA	200 nM × 20 min				↓ (≈35.7 ± 5%) SEM	Na et al., 2025 [12]
0.2 mM	16 h	BSA	No		↔ (≈55.6 ± 5%) SEM		↓ (≈78.9 ± 5%) SEM	Liu et al., 2015 [23]
0.2 mM	16 h	BSA	200 nM × 30 min		↓ (≈21.8 ± 5%) SEM		↓ (≈70.4 ± 5%) SEM	Liu et al., 2015 [23]
0.2 mM	16 h	BSA	100 nM × 120 min		↓VC			Kirk-Ballard et al., 2014 [24]
0.2 mM	16 h	BSA	50 nM × 15 min		↓ (≈7.8 ± 5%)			Gone et al., 2024 [25]
0.2 mM	16 h	BSA	17.2 nM × 15 min		↓ (≈17.5 ± 5%)			De Wilde et al., 2010 [7]
0.2 mM	16 h	NR	10 nM × 30 min				↓ (≈44.2 ± 5%) SEM	Jheng et al., 2012 [26]
0.2 mM	16 h	EtOH	No				↔ (≈97.1 ± 5%)	Parvaneh et al., 2010 [27]
0.2 mM	16 h	EtOH	100 nM × 30 min				↔ (≈100.0 ± 5%)	Parvaneh et al., 2010 [27]
0.2 mM	19 h	BSA	10 nM × 10 min		↓ VC			Blackburn et al., 2020 [28]
0.2 mM	19 h	BSA	100 nM × 10 min		↓ VC			Blackburn et al., 2020 [28]
0.2 mM	24 h	BSA	No				↔ (≈103.4 ± 5%)	Tatebe et al., 2011 [29]
0.2 mM	24 h	BSA	100 nM × 10 min		↔ (≈88.4 ± 5%)	↔ (≈105.9 ± 5%)	↔ (≈100.0 ± 5%)	Tatebe et al., 2011 [29]
0.2 mM	24 h	BSA	10 nM × 30 min		↓ (≈22.8 ± 5%)	↓ (≈56.8 ± 5%)		Norouzi et al., 2019 [15]
0.2 mM	24 h	BSA	10 nM × 3 min	↓ (≈21.0 ± 5%)	↓ (≈10.5 ± 5%)		↓ (≈47.7 ± 5%)	Gwon et al., 2024 [30]
0.2 mM	24 h	BSA	No		↔ (≈96.9 ± 5%)			Wu et al., 2024 [31]
0.2 mM	24 h	BSA	Yes, unknown conditions		↓ (≈59.6 ± 5%)		↓ (≈73.6 ± 5%)	Wu et al., 2024 [31]
0.2 mM	24 h	BSA	50 nM × 10 min	↓ (≈45.7 ± 5%)	↓ (≈57.1 ± 5%)		↓ (≈43.5 ± 5%)	Sun et al., 2024 [32]
0.2 mM	24 h	BSA	100 nM × 10 min		↓ (≈47.7 ± 5%) SEM		↓ (≈66.2 ± 5%) SEM	Li et al., 2023 [33]
0.2 mM	24 h	BSA	10 nM × 3 min	↓ (≈20.9 ± 5%)	↓ (≈27.1 ± 5%)		↓ (≈43.6 ± 5%)	Oh et al., 2022 [34]
0.2 mM	24 h	BSA	10 nM × 3 min	↓ (≈38.0 ± 5%) SEM	↓ (≈17.5 ± 5%) SEM		↓ (≈42.1 ± 5%) SEM	Jung et al., 2022 [35]
0.2 mM	24 h	BSA	10 nM × 3 min	↓ (≈30.6 ± 10%) SEM	↓ (≈26.5 ± 10%) SEM		↓ (≈52.5 ± 5%) SEM	Park et al., 2021 [36]
0.2 mM	24 h	BSA	No		↔ (≈72.7 ± 5%)			Pesta et al., 2021 [37]
0.2 mM	24 h	BSA	100 nM × 10 min		↓ (≈57.6 ± 5%)			Pesta et al., 2021 [37]
0.2 mM	24 h	BSA	10 nM × 3 min	↓ (≈24.0 ± 5%) SEM	↓ (≈38.8 ± 5%) SEM		↓ (≈40.3 ± 5%) SEM	Jung et al., 2021 [38]
0.2 mM	24 h	BSA	10 nM × 3 min	↓ (≈50.6 ± 5%) SEM	↓ (≈23.3 ± 5%) SEM		↓ (≈44.2 ± 5%) SEM	Kim et al., 2021 [39]
0.2 mM	24 h	BSA	100 nM × 30 min	↓ (≈46.9 ± 5%)	↓ (≈78.8 ± 5%)		↓ (≈69.3 ± 5%)	Liu et al., 2020 [40]
0.2 mM	24 h	BSA	100 nM × 10 min			↓ (≈81.9 ± 5%)		Chiu et al., 2021 [41]
0.2 mM	24 h	BSA	100 nM × 30 min	↓ (≈45.0 ± 5%)	↓ (≈36.4 ± 5%)		↓ (≈59.4 ± 5%)	Chuang et al., 2020 [42]
0.2 mM	24 h	BSA	Unknown × 15 min	↓ (≈33.3 ± 5%) SEM	↓ (≈52.4 ± 5%)			Park et al., 2020 [43]
0.2 mM	24 h	BSA	10 nM × 3 min	↓ (≈43.8 ± 5%) SEM	↓ (≈17.6 ± 5%) SEM		↓ (≈30.3 ± 5%) SEM	Sun et al., 2020 [44]
0.2 mM	24 h	BSA	10 nM × 3 min	↓ (≈6.0 ± 5%) SEM	↓ (≈20.8 ± 5%) SEM		↓ (≈34.1 ± 5%) SEM	Jung et al., 2017 [45]
0.2 mM	24 h	BSA	Unknown		↓ (≈76.8 ± 5%) SEM	↓ (≈57.7 ± 5%) SEM	↓ (≈51.9 ± 5%) SEM	Kulabas et al., 2018 [46]
0.2 mM	24 h	BSA	100 nM × 15 min		↓ VC	↔ (≈57.5 ± 5%) SEM	↓ (≈85.1 ± 5%) SEM	Yang et al., 2013 [47]
0.2 mM	24 h	BSA	10 nM × 3 min	↓ (≈48.8 ± 5%) SEM	↓ (≈33.3 ± 5%) SEM			Jung et al., 2015 [48]
0.2 mM	24 h	BSA	10 nM × 3 min	↓ (≈75.8 ± 5%) SEM	↓ (≈68.0 ± 5%) SEM		↓ (≈42.3 ± 5%) SEM	Jung et al., 2017 [49]
0.2 mM	24 h	BSA	No				↔ (≈141.3 ± 5%) SEM	Zhang et al., 2015 [19]
0.2 mM	24 h	BSA	100 nM × 30 min			↓ (≈60.4 ± 5%)	↓ (≈82.3 ± 5%)	Zhang et al., 2015 [19]
0.2 mM	24 h	BSA	No		↓ (≈46.1 ± 5%) SEM			Yang et al., 2012 [50]
0.2 mM	48 h	BSA	10 nM × 3 min	↓ (≈24.3 ± 5%) SEM	↓ (≈19.5 ± 5%) SEM		↓ (≈40.9 ± 5%) SEM	Pyun et al., 2021 [51]
0.2 mM	24 h	BSA	100 nM × 30 min		↓ (≈58.8 ± 5%) SEM			Yang et al., 2012 [50]
0.2 mM	48 h	BSA	10 nM × 3 min	↓ (≈24.3 ± 5%)	↓ (≈18.3 ± 5%)		↓ (≈41.0 ± 5%)	Jung et al., 2020 [52]
0.2 mM	48 h	BSA	No		↔ VC		↔ (≈104.1 ± 5%)	de Figueiredo et al., 2015 [53]
0.2 mM	48 h	BSA	100 nM × 30 min		↓ (≈50.0 ± 5%)		↓ (≈67.3 ± 5%)	de Figueiredo et al., 2015 [53]
0.25 mM	12 h	BSA	100 nM × 30 min				↔ (≈95.3 ± 5%)	Zhao et al., 2012 [54]
0.25 mM	16 h	BSA	100 nM × 30 min		↓ (≈46.6 ± 5%)			Kim et al., 2022 [55]
0.25 mM	16 h	BSA	20 nM × 60 min (with amino acids)		↓ (≈48.4 ± 5%) SEM			Cruz et al., 2020 [56]
0.25 mM	16 h	BSA	No		↔ (≈100.0 ± 5%) SEM			Bosquet et al., 2018 [57]
0.25 mM	16 h	BSA	100 nM × 10 min		↓ (≈50.4 ± 5%) SEM			Bosquet et al., 2018 [57]
0.25 mM	16 h	Unconjugated	100 nM × 30 min				↔ (≈100.0 ± 5%) SEM	Lee et al., 2017 [58]
0.25 mM	16 h	BSA	100 nM × 30 min				↔ (≈78.4 ± 5%) SEM	Lee et al., 2017 [58]
0.25 mM	16 h	BSA	No		↓ (≈74.0 ± 5%) SEM			Zhou et al., 2007 [59]
0.25 mM	16 h	BSA	100 nM × 10 min		↓ (≈50.0 ± 5%) SEM			Hage Hassan et al., 2012 [60]
0.25 mM	16 h	BSA	No				↔ (≈96.8 ± 5%)	Zhang et al., 2010 [61]
0.25 mM	16 h	BSA	100 nM × 20 min				↔ (≈96.0 ± 5%)	Zhang et al., 2010 [61]
0.25 mM	16 h	BSA	100 nM × 10 min		↓ VC			Chavez et al., 2005 [11]
0.25 mM	16 h	BSA	No				↔ (≈100.0 ± 5%) SEM	Feng et al., 2012 [20]
0.25 mM	16 h	BSA	100 nM × 30 min				↓ (≈87.4 ± 5%) SEM	Feng et al., 2012 [20]
0.25 mM	18 h	BSA	No				↓ (≈61.8 ± 5%)	Park et al., 2014 [13]
0.25 mM	18 h	BSA	100 nM × 10 min		↓ (≈57.8 ± 5%)		↓ (≈42.7 ± 5%)	Park et al., 2014 [13]
0.25 mM	18 h	BSA	100 nM × 30 min		↔ VC		↓ (≈65.1 ± 5%) SEM	Qin et al., 2009 [62]
0.25 mM	24 h	BSA	No		↓ VC		↓ (≈65.6 ± 5%) SEM	Cheon et al., 2014 [8]
0.25 mM	24 h	BSA	100 nM × 30 min		↓ VC		↓ (≈54.8 ± 5%) SEM	Cheon et al., 2014 [8]
0.25 mM	24 h	BSA			↓ (≈61.2 ± 5%) SEM			Chang et al., 2018 [63]
0.25 mM	24 h	BSA	100 nM × 30 min	↔ (≈112.5 ± 5%)	↔ (≈78.0 ± 5%)	↔ (≈85.0 ± 5%)	↔ (≈113.6 ± 5%)	Pan et al., 2023 [16]
0.25 mM	24 h	BSA	100 nM × 30 min	↔ (≈94.7 ± 5%) SEM	↓ (≈90.5 ± 5%) SEM		↓ VC	Li et al., 2023 [64]
0.25 mM	24 h	BSA	100 nM × 30 min		↓ (≈55.9 ± 5%) SEM	↓ (≈68.8 ± 5%) SEM	↓ (≈72.7 ± 5%) SEM	Yu et al., 2022 [65]
0.25 mM	24 h	BSA	No				↓ (≈50.0 ± 5%) SEM	Jiao et al., 2022 [66]
0.25 mM	24 h	BSA	100 nM × unknown				↓ (≈38.6 ± 5%) SEM	Jiao et al., 2022 [66]
0.25 mM	24 h	BSA	No		↓ (≈54.5 ± 5%)			He et al., 2022 [67]
0.25 mM	24 h	BSA	100 nM × 60 min				↓ (≈47.8 ± 5%) SEM	Hu et al., 2022 [68]
0.25 mM	24 h	BSA	No		↔ (≈100.0 ± 5%)			Nan et al., 2021 [69]
0.25 mM	24 h	BSA	100 nM × 30 min		↓ (≈45.7 ± 5%)			Nan et al., 2021 [69]
0.25 mM	24 h	BSA	No	↓ (≈32.2 ± 5%)	↔ (≈70.9 ± 5%)			Shen et al., 2019 [70]
0.25 mM	24 h	BSA	100 nM × 30 min	↓ (≈27.2 ± 5%)	↓ (≈30.3 ± 5%)		↓ (≈51.4 ± 5%)	Shen et al., 2019 [70]
0.25 mM	24 h	BSA	100 nM × 30 min		↓ (≈60.4 ± 5%)	↓ (≈55.3 ± 5%)	↓ VC	Zhang et al., 2019 [71]
0.25 mM	24 h	BSA	No				↔ (≈109.0 ± 5%)	Fujiwara et al., 2017 [72]
0.25 mM	24 h	BSA	100 nM × 20 min				↓ (≈47.2 ± 5%)	Fujiwara et al., 2017 [72]

NOTES: NR indicates detail was not reported. VC indicates visual confirmation was used and statistical comparisons were not made for relevant groups or only used single image densitometry. Abbreviations: bovine serum albumin (BSA), ethanol (EtOH), low glucose (LG), high glucose (HG). ↓: indicates a reported decrease in the target versus control; ↔: indicates no difference reported in the target versus control.

**Table 2:** Findings from systematic search results on the effect of moderately greater than pharmacokinetically attainable levels of palmitate treatment at various concentrations for various durations on common indicators of insulin sensitivity.

Palmitate Concentration	Duration	Vehicle	Insulin Stimulation	Insulin Receptor Substrate (pIRS)	Protein Kinase B (pAkt)	Glucose Transporter 4 (GLUT4)	Glucose Uptake	Reference
0.3 mM	12 h	BSA	Unknown		↓ VC			Chen et al., 2016 [10]
0.3 mM	16 h	BSA	No		↔ (≈75.0 ± 5%) SEM			Lee et al., 2020 [73]
0.3 mM	16 h	BSA	100 nM × 120 min		↓ (≈84.6 ± 5%) SEM			Lee et al., 2020 [73]
0.3 mM	16 h	BSA	No				↓ (≈38.4 ± 5%) SEM	Chien et al., 2020 [74]
0.3 mM	18 h	BSA	100 nM × 15 min		↓ (≈61.7 ± 5%)			Smimmo et al., 2025 [75]
0.3 mM	24 h	BSA	No		↓ (≈36.3 ± 5%)			Yang et al., 2024 [76]
0.3 mM	24 h	BSA	No	↓ (≈46.9 ± 5%)	↓ (≈65.7 ± 5%)			Mao et al., 2025 [77]
0.3 mM	24 h	BSA	10 nM × 30 min		↓ (≈25.7 ± 5%)	↓ (≈36.3 ± 5%)		Norouzi et al., 2019 [15]
0.3 mM	24 h *	BSA	20 nM × 15 min		↓ (≈56.8 ± 5%)			Kwon et al., 2015 [78]
0.35 mM	18 h	BSA	No		↔ (≈92.8 ± 5%) SEM			Chen et al., 2017 [79]
0.35 mM	18 h	BSA	10 nM × 10 min		↓ (≈50.0 ± 5%) SEM		↓ (≈78.1 ± 5%) SEM	Chen et al., 2017 [79]
0.375 mM	16 h	BSA	100 nM × 30 min		↓ (≈59.1 ± 5%) SEM			Tardif et al., 2014 [80]
0.4 mM	6 h	BSA	No				↔ (≈89.2 ± 5%)	Dai et al., 2016 [81]
0.4 mM	6 h	BSA	1 µM × 18 min				↓ (≈71.4 ± 5%)	Dai et al., 2016 [81]
0.4 mM	24 h	BSA	100 nM × 30 min		↓ (≈67.6 ± 5%)			Dai et al., 2016 [81]
0.4 mM	12 h	BSA	Unknown		↓ VC			Chen et al., 2016 [10]
0.4 mM	16 h	BSA	No		↔ (≈50.0 ± 5%)		↔ (≈79.2 ± 5%) SEM	Lee et al., 2020 [73]
0.4 mM	16 h	BSA	100 nM × 120 min		↓ (≈66.1 ± 5%) SEM		↓ (≈66.6 ± 5%) SEM	Lee et al., 2020 [73]
0.4 mM	16 h	BSA	17.2 nM × 15 min		↓ (≈15.2 ± 5%)			De Wilde et al., 2010 [7]
0.4 mM	18 h	BSA	100 nM × 15 min		↓ (≈53.0 ± 5%)	↓ (≈63.6 ± 5%)		D’Souza et al., 2020 [82]
0.4 mM	24 h	BSA	No			↓ (≈59.5 ± 5%)	↓ (≈79.6 ± 5%)	Zhang et al., 2024 [83]
0.4 mM	24 h	BSA	100 nM × 30 min				↓ (≈85.5 ± 5%)	Zhang et al., 2024 [83]
0.4 mM	24 h	BSA	No		↔ (≈66.6 ± 5%) SEM			Jakovljevic et al., 2021 [84]
0.4 mM	24 h	BSA	120 nM × 15 min		↓ (≈47.6 ± 5%) SEM			Jakovljevic et al., 2021 [84]
0.4 mM	24 h	BSA	100 nM × 10 min			↓ (≈58.3 ± 5%)	↓ (≈51.6 ± 5%)	Chiu et al., 2021 [41]
0.4 mM	24 h	BSA	100 nM × 30 min	↓ (≈38.5 ± 5%)	↓ (≈68.8 ± 5%)		↓ (≈60.0 ± 5%)	Liu et al., 2020 [40]
0.4 mM	24 h	BSA	100 nM × 30 min			↓ VC	↓ VC	Wu et al., 2018 [85]
0.4 mM	24 h	BSA	100 nM × 15 min		↓ VC	↓ (≈30.3 ± 5%) SEM	↔ (≈75.5 ± 5%) SEM	Yang et al., 2013 [47]
0.4 mM	24 h	BSA	No		↓ (≈30.7 ± 5%) SEM			Yang et al., 2012 [50]
0.4 mM	24 h	BSA	100 nM × 30 min		↓ (≈38.8 ± 5%) SEM			Yang et al., 2012 [50]
0.4 mM	36 h	BSA	No	(≈167.1 ± 5%) SEM				Fan et al., 2021 [86]
0.5 mM	0.5 h	BSA	Unknown		↔ VC			Chen et al., 2016 [10]
0.5 mM	1 h	BSA	Unknown		↔ VC			Chen et al., 2016 [10]
0.5 mM	2 h	BSA	Unknown		↔ VC			Chen et al., 2016 [10]
0.5 mM	4 h	BSA	No				↑ (≈50.0 ± 5%)	Nieuwoudt et al., 2017 [87]
0.5 mM	4 h	BSA	1 µM × 30 min				↓ (≈71.8 ± 5%)	Nieuwoudt et al., 2017 [87]
0.5 mM	4 h	BSA	1 µM × 10 min			↔ (≈114.8 ± 5%)		Nieuwoudt et al., 2017 [87]
0.5 mM	4 h	BSA	Unknown		↓ VC			Chen et al., 2016 [10]
0.5 mM	6 h	BSA	No		↑ (≈1060.0 ± 5%)		↔ (≈94.8 ± 5%)	Kim et al., 2018 [88]
0.5 mM	6 h	BSA	100 nM × 15 min		↓ (≈58.0 ± 5%)		↓ (≈73.4 ± 5%)	Kim et al., 2018 [88]
0.5 mM	6 h	BSA	Unknown		↓ VC			Chen et al., 2016 [10]
0.5 mM	8 h	BSA	Unknown		↓ VC			Chen et al., 2016 [10]
0.5 mM	10 h	BSA	Unknown		↓ VC			Chen et al., 2016 [10]
0.5 mM	12 h	BSA	No		↔ VC			Chen et al., 2016 [10]
0.5 mM	12 h	BSA	Unknown		↓ VC			Chen et al., 2016 [10]
0.5 mM	12 h	BSA	No		↓ VC			Li et al., 2011 [89]
0.5 mM	12 h	BSA	100 nM × 5 min		↓ VC			Li et al., 2011 [89]
0.5 mM	12 h	BSA	No	↔ (100.0 ± 5%)	↔ (100.0 ± 5%)			Zhao et al., 2012 [54]
0.5 mM	12 h	BSA	100 nM × 30 min	↑ (≈356.5 ± 5%)	↓ (≈52.9 ± 10%)		↓ (≈68.4 ± 5%)	Zhao et al., 2012 [54]
0.5 mM	16 h	BSA	100 nM × 60 min			↓ VC	↓ (≈26.2 ± 5%) SEM	Zhou et al., 2014 [90]
0.5 mM	16 h	BSA	No		↔ (≈100.0 ± 5%) SEM			Paez et al., 2023 [91]
0.5 mM	16 h	BSA	100 nM × 120 min		↓ (≈55.1 ± 5%) SEM			Paez et al., 2023 [91]
0.5 mM	16 h	BSA	100 nM × 30 min	↑ (110.0 ± 5%) SEM	↓ (77.1 ± 5%) SEM		↓ (84.3 ± 5%) SEM	Jia et al., 2021 [92]
0.5 mM	16 h	BSA	100 ng/mL × 15 min		↓ (≈36.2 ± 10%)			Rustamov et al., 2024 [93]
0.5 mM	16 h	BSA	20 nM × 60 min (with amino acids)		↓ (≈25.7 ± 5%) SEM			Cruz et al., 2020 [56]
0.5 mM	16 h	BSA	100 nM × 10 min		↓ (≈58.6 ± 5%)			Pinel et al., 2018 [94]
0.5 mM	16 h	BSA	1 µg/mL × 4 h		↓ (≈76.8 ± 5%)			Pinel et al., 2018 [94]
0.5 mM	16 h	BSA	100 nM × 10 min	↑ (≈356.5 ± 5%)	↓ (≈27.7 ± 5%)			Aoki et al. [95]
0.5 mM	16 h	Unconjugated	100 nM × 30 min				↔ (≈100.0 ± 5%) SEM	Botteri et al., 2018 [96]
0.5 mM	16 h	BSA	100 nM × 30 min				↓ (≈56.8 ± 5%) SEM	Lee et al., 2017 [58]
0.5 mM	16 h	BSA	No	↔ (≈125.0 ± 5%)	↔ (≈66.6 ± 5%)	↔ (≈90.0 ± 5%)		Lee et al., 2017 [58]
0.5 mM	16 h	BSA	100 nM × 10 min	↑ (≈200.0 ± 5%)	↓ (≈42.8 ± 5%)	↓ (≈63.7 ± 5%)		Li et al., 2018 [97]
0.5 mM	16 h	BSA	No		↔ (≈83.3 ± 5%)			Li et al., 2018 [97]
0.5 mM	16 h	BSA	Yes, unknown conditions		↓ (≈63.6 ± 5%)			Qin et al., 2017 [98]
0.5 mM	16 h	BSA	100 nM × 30 min	↑ VC	↓ VC		↓ (≈61.1 ± 5%) SEM	Qin et al., 2017 [98]
0.5 mM	16 h	BSA	100 nM × 10 min		↓ (≈62.9 ± 5%)			Lee et al., 2017 [58]
0.5 mM	16 h	BSA	1 nM × 15 min		↓ VC			Capel et al., 2016 [99]
0.5 mM	16 h	BSA	100 nM × 15 min		↓ Thr308 (≈54.0 ± 5%)			Capel et al., 2016 [99]
0.5 mM	16 h	BSA	100 nM × 15 min		↓ Ser473 (≈60.0 ± 5%)		↓ (≈80.7 ± 5%)	Park et al., 2016 [100]
0.5 mM	16 h	BSA	100 nM × 10 min		↓ Thr308 (≈34.3 ± 5%)			Pinel et al., 2015 [101]
0.5 mM	16 h	BSA	100 nM × 10 min		↓ Ser473 (≈61.667 ± 5%)		↓ (≈56.0 ± 5%)	Capel et al., 2015 [102]
0.5 mM	16 h	BSA	100 nM × >10 min		↓ (≈75.0 ± 5%)		↓ (≈56.6 ± 5%)	Salvado et al., 2013 [103]
0.5 mM	16 h	BSA	100 nM × 30 min			↓ (≈32.2 ± 5%)	↓ (≈51.5 ± 5%)	Jing et al., 2017 [104]
0.5 mM	16 h	BSA	No				↔ (≈86.2 ± 5%)	Karimfar et al., 2015 [105]
0.5 mM	16 h	BSA	100 nM × 30 min				↓ (≈51.0 ± 5%)	Karimfar et al., 2015 [105]
0.5 mM	16 h	BSA	No	↑ (≈193.7 ± 5%) SEM	↓ (≈80.0 ± 5%) SEM			Zhou et al., 2007 [59]
0.5 mM	16 h	BSA	100 nM × 10 min	↓ (≈81.9 ± 5%)	↓ (≈72.5 ± 5%)		↓ (≈75.6 ± 5%)	Bakhtiyari et al. [106]
0.5 mM	16 h	BSA	No				↔ (≈87.5 ± 5%)	Zhang et al., 2010 [61]
0.5 mM	16 h	BSA	100 nM × 20 min				↓ (≈85.3 ± 5%)	Zhang et al., 2010 [61]
0.5 mM	16 h	BSA	No			↓ (≈236.3 ± 5%)	↔ (≈154.3 ± 5%)	Jove et al., 2005 [107]
0.5 mM	16 h	BSA	100 nM × 30 min				↓ (≈65.3 ± 5%)	Jove et al., 2005 [107]
0.5 mM	16 h	BSA	No		↔ (83.3 ± 5%) SEM			Henique et al., 2010 [108]
0.5 mM	16 h	BSA	100 nM × 10 min		↓ (49.1 ± 5%) SEM			Henique et al., 2010 [108]
0.5 mM	16 h	BSA	No	↑ VC				Coll et al., 2010 [109]
0.5 mM	16 h	BSA	100 nM × 10 min		↓ (≈78.9 ± 5%)		↓ (≈61.3 ± 5%)	Coll et al., 2010 [109]
0.5 mM *	16 h	BSA	No		↓ (≈43.1 ± 5%)			Zhou et al., 2007 [59]
0.5 mM	16 h	BSA	100 nM × 10 min		↓ (≈79.5 ± 5%) SEM			Hage Hassan et al., 2012 [60]
0.5 mM	16 h	BSA	No		↔ (≈103.2 ± 5%) SEM		↔ (≈100.0 ± 5%) SEM	Feng et al., 2012 [20]
0.5 mM	16 h	BSA	100 nM × 30 min		↓ (≈60.4 ± 5%) SEM		↓ (≈80.0 ± 5%) SEM	Feng et al., 2012 [20]
0.5 mM	16 h	BSA	No				↔ (≈101.5 ± 5%) SEM	Feng et al., 2012 [110]
0.5 mM	16 h	BSA	100 nM × 20 min				↓ (≈82.8 ± 5%) SEM	Feng et al., 2012 [110]
0.5 mM	16 h	BSA	No				↔ (≈97.3 ± 5%) SEM	Gorgani-Firuzjaee et al., 2012 [111]
0.5 mM	16 h	BSA	100 nM × 30 min				↓ (76.0 ± 5%) SEM	Gorgani-Firuzjaee et al., 2012 [111]
0.5 mM	16 h	BSA	100 nM × 15 min	↓ (≈70.4 ± 5%) SEM	↓ (≈68.7 ± 5%) SEM			Gorgani-Firuzjaee et al., 2012 [111]
0.5 mM	18 h	BSA	No				↓ (≈41.8 ± 5%)	Park et al., 2014 [13]
0.5 mM	18 h	BSA	100 nM × 10 min		↓ (≈50.5 ± 5%)		↓ (≈23.9 ± 5%)	Park et al., 2014 [13]
0.5 mM	18 h	BSA	100 nM × 30 min		↓ (≈58.1 ± 5%)	↓ (≈62.5 ± 5%)	↓ (≈61.5 ± 5%)	Li et al., 2024 [112]
0.5 mM	18 h	No	10 nM × 20 min		↓ (≈66.6 ± 5%) SEM			Aswad et al., 2014 [113]
0.5 mM	18 h	BSA	10 nM × 20 min		↓ (≈65.4 ± 5%) SEM			Aswad et al., 2014 [113]
0.5 mM	19 h	BSA	10 nM × 10 min		↓ VC			Blackburn et al., 2020 [28]
0.5 mM	19 h	BSA	100 nM × 10 min		↓ VC			Blackburn et al., 2020 [28]
0.5 mM	24 h	EtOH	100 nM × 30 min		↔ (≈91.0 ± 5%)		↔ (≈86.6 ± 5%)	Rivera et al., 2021 [114]
0.5 mM	24 h	DMSO	No	↓ (≈83.8 ± 5%)	↑ (≈134.4 ± 5%)		↓ (≈81.4 ± 5%)	Wu et al., 2020 [115]
0.5 mM	24 h	BSA	100 nM × 24 h		↓ (≈43.2 ± 5%)	↓ (≈66.6 ± 5%)		Song et al., 2024 [116]
0.5 mM	24 h	BSA	100 nM × 15 min		↓VC	↓VC	↑ (≈231.5 ± 5%)	Shree et al., 2016 [117]
0.5 mM	24 h	BSA	No				↓ (≈72.6 ± 5%)	Li et al., 2022 [118]
0.5 mM	24 h	BSA	1 µg/mL × 30 min				↓ (≈62.6 ± 5%)	Li et al., 2022 [118]
0.5 mM	24 h	BSA	No		↓VC		↓ (≈67.1 ± 5%) SEM	Cheon et al., 2014 [8]
0.5 mM	24 h	BSA	100 nM × 30 min		↓VC		↓ (≈52.4 ± 5%) SEM	Cheon et al., 2014 [8]
0.5 mM	24 h	BSA	No		↔ (≈72.7 ± 5%)			Silva et al., 2024 [119]
0.5 mM	24 h	BSA	100 nM × 30 min		↓ (≈39.2 ± 5%)			Silva et al., 2024 [119]
0.5 mM	24 h	BSA	No		↓ VC			Li et al., 2024 [120]
0.5 mM	24 h	BSA	100 nM × 30 min		↓ (≈59.5 ± 5%)		↓ (≈34.9 ± 5%)	Li et al., 2024 [120]
0.5 mM	24 h	BSA	100 nM × 30 min	↑ (≈204.1 ± 5%)	↓ (≈46.3 ± 5%)	↓ (≈30.0 ± 5%)	↑ (≈140.9 ± 5%)	Pan et al., 2023 [16]
0.5 mM	24 h	BSA	No				↓ (≈91.5 ± 5%) SEM	Guo et al., 2023 [121]
0.5 mM	24 h	BSA	1 µM × 60 min		↓ (≈77.7 ± 5%) SEM			Guo et al., 2023 [121]
0.5 mM	24 h	BSA	No	↔ (≈81.2 ± 5%) SEM	↔ (≈80.0 ± 5%) SEM		↔ (≈78.2 ± 5%) SEM	Eo et al., 2022 [122]
0.5 mM	24 h	BSA	50 nM × 15 min	↔ (≈68.4 ± 5%) SEM	↓ (≈45.2 ± 5%) SEM		↓ (≈64.7 ± 5%) SEM	Eo et al., 2022 [122]
0.5 mM	24 h	BSA	No		↔ Thr308 (≈138.4 ± 5%) SEM			Rios-Morales et al., 2022 [123]
0.5 mM	24 h	BSA	No		↔ Ser473(≈70.0 ± 5%) SEM			Rios-Morales et al., 2022 [123]
0.5 mM	24 h	BSA	100 nM × 20 min		↓ (≈71.4 ± 5%) SEM			Rios-Morales et al., 2022 [123]
0.5 mM	24 h	BSA	No		↔ (≈85.7 ± 5%) SEM			Li et al., 2021 [124]
0.5 mM	24 h	BSA	100 nM × 20 min		↓ (≈38.8 ± 5%) SEM			Li et al., 2021 [124]
0.5 mM	24 h	BSA	7 nM × 15 min		↓ (≈21.9 ± 5%)			Rodrigues et al., 2021 [125]
0.5 mM	24 h	BSA	200 nM × 20 min	↓ (≈51.0 ± 5%)	↓ (≈67.9 ± 5%)			Munoz et al., 2021 [126]
0.5 mM	24 h	BSA	No			↓ (≈42.3 ± 5%)	↓ (≈63.3 ± 5%)	Guo 2021 et al. [127]
0.5 mM	24 h	BSA	10 nM × 15 min	↓ (≈80.0 ± 5%)	↓ (≈69.3 ± 5%)			Luo et al., 2020 [128]
0.5 mM	24 h	BSA	100 nM × unknown		↓ (≈65.3 ± 5%)		↓ (≈54.0 ± 5%)	Chen et al., 2019 [129]
0.5 mM	24 h	BSA	No		↓ (≈63.1 ± 5%)			Guo et al., 2020 [130]
0.5 mM	24 h	BSA	1 µM × 60 min		↓ (≈75.4 ± 5%)			Guo et al., 2020 [130]
0.5 mM	24 h	BSA	unknown		↓ (≈47.3 ± 5%)			Liu et al., 2019 [131]
0.5 mM	24 h	BSA	100 nM × unknown	↓ (≈52.8 ± 5%)	↓ (≈30.1 ± 5%)			Bakhtiyari et al., 2019 [132]
0.5 mM	24 h	BSA	No	↓ (≈61.3 ± 5%)		↓ (≈55.0 ± 5%)		Zhang et al., 2018 [17]
0.5 mM	24 h	BSA	10 nM × 30 min		↓ (≈51.2 ± 5%)			Huang et al., 2017 [133]
0.5 mM	24 h	BSA	100 nM × 10 min		↔ VC			Dziewulska et al., 2012 [134]
0.5 mM	24 h	BSA	100 nM × 10 min		↓ (≈17.9 ± 5%)			Dziewulska et al., 2012 [134]
0.5 mM	18 h + 9 h	No	10 nM × 20 min		↓ (≈0.00 ± 5%)			Aswad et al., 2014 [113]
0.5 mM	18 h + 9 h	BSA	10 nM × 20 min		↓ (≈25.0 ± 5%)			Aswad et al., 2014 [113]
0.5 mM	48 h	BSA	100 nM × 30 min		↓ (≈45.4 ± 5%) SEM		↓ (≈55.5 ± 5%)	Yoon et al., 2021 [135]

NOTES: * indicates aspects of results were repeated with separate experiment within the same report using similar treatment conditions. NR indicates detail was not reported. VC indicates visual confirmation was used and statistical comparisons were not made for relevant groups or only used single image densitometry. The addition sign (+) following a treatment duration indicates a recovery time following palmitate treatment. Abbreviations: bovine serum albumin (BSA) and ethanol (EtOH). ↓: indicates a reported decrease in the target versus control; ↔: indicates no difference reported in the target versus control; ↑: indicates a reported increase in the target versus control.

**Table 3:** Findings from systematic search results on the effect of far greater than pharmacokinetically attainable (proof-of-concept) levels of palmitate treatment at various concentrations for various durations on common indicators of insulin sensitivity.

Palmitate Concentration	Duration	Vehicle	Insulin Stimulation	Insulin Receptor Substrate (pIRS)	Protein Kinase B (pAkt)	Glucose Transporter 4 (GLUT4)	Glucose Uptake	Reference
0.6 mM	24 h	BSA	No		↔ (≈75.4 ± 5%) SEM			Lee et al., 2022 [136]
0.6 mM	24 h	BSA	100 nM × 15 min		↓ (≈0.00 ± 5%)		↓ (≈62.5 ± 5%)	Lee et al., 2022 [136]
0.6 mM	24 h	BSA	100 nM × 15 min		↓ VC	↓ (≈21.2 ± 5%) SEM	↓ (≈73.4 ± 5%) SEM	Yang et al., 2013 [47]
0.6 mM	24 h	BSA	No		↓ (≈21.7 ± 5%)			Abe et al., 2016 [137]
0.6 mM	24 h	BSA	100 nM × 60 min		↓ (≈37.6 ± 5%)			Abe et al., 2016 [137]
0.6 mM	24 h	BSA	No		↓ (≈30.7 ± 5%) SEM			Yang et al., 2012 [50]
0.6 mM	24 h	BSA	100 nM × 30 min		↓ (≈29.4 ± 5%) SEM			Yang et al., 2012 [50]
0.6 mM	48 h	BSA	unknown	↓ (≈24.4 ± 5%)				Tang et al., 2018 [138]
0.7 mM	18 h	BSA	No		↔ (≈100.0 ± 5%)			Rieusset et al., 2012 [139]
0.7 mM	18 h	BSA	10 nM × 20 min		↓ (≈29.2 ± 5%)			Rieusset et al., 2012 [139]
0.75 mM	1 h	BSA	100 nM × 10 min		↓ VC			Hage Hassan et al., 2012 [60]
0.75 mM	2 h	BSA	No	↑ (≈266.6 ± 5%)	↔ (≈100.0 ± 5%)		↓ (≈71.7 ± 5%)	Schmitz-Peiffer et al., 1999 [4]
0.75 mM	2 h	BSA	100 nM × 10 min	↔ (≈93.7 ± 5%)	↓ (≈58.9 ± 5%)		↓ (≈77.1 ± 5%)	Schmitz-Peiffer et al., 1999 [4]
0.75 mM	2 h	BSA	100 nM × 10 min		↓ VC			Hage Hassan et al., 2012 [60]
0.75 mM	4 h	BSA	100 nM × 10 min		↓ VC			Hage Hassan et al., 2012 [60]
0.75 mM	5 h	BSA	No	↑ (≈268.5 ± 5%)	↔ VC			Deng et al., 2012 [140]
0.75 mM	5 h	BSA	100 nM × 10 min	↓ (≈58.1 ± 5%)	↔ VC			Deng et al., 2012 [140]
0.75 mM	6 h	BSA	10 nM × 10 min		↔ (≈85.7 ± 5%)			De Hart et al., 2023 [141]
0.75 mM	6 h	BSA	10 nM × 10 min		↓ VC			Wang et al., 2009 [142]
0.75 mM	8 h	BSA	100 nM × 60 min			↓(≈54.5 ± 5%)	↓(≈45.1 ± 5%)	Samra et al., 2024 [143]
0.75 mM	12 h	BSA	10 nM × 10 min		↔ (≈71.4 ± 5%)			De Hart et al., 2023 [141]
0.75 mM	12 h	BSA	10 nM × 10 min		↓ VC			Wang et al., 2009 [142]
0.75 mM	16 h	EtOH	No				↔ (≈92.5 ± 5%)	Parvaneh et al., 2010 [27]
0.75 mM	16 h	EtOH	100 nM × 30 min				↓ (≈71.6 ± 5%)	Parvaneh et al., 2010 [27]
0.75 mM	16 h	BSA	No		↓ (≈43.8 ± 5%)	↓ (≈32.5 ± 5%)	↓ (≈61.7 ± 5%)	Yoon et al., 2018 [144]
0.75 mM	16 h	BSA	1 µM × unknown		↓ (≈44.6 ± 5%)		↓ (≈74.5 ± 5%)	Yoon et al., 2018 [144]
0.75 mM	16 h	BSA	100 nM × 15 min	↓ (≈80.8 ± 5%) SEM	↓ (≈67.6 ± 5%) SEM			Hsieh et al., 2014 [145]
0.75 mM	16 h	BSA	100 nM × 10 min		↓ VC			Bandet et al., 2023 [146]
0.75 mM	16 h	BSA	100 nM × 30 min		↓ (≈56.8 ± 5%)	↓ (≈62.0 ± 5%)		Lin et al., 2017 [147]
0.75 mM	16 h	BSA	100 nM × 15 min		↓ (≈53.2 ± 5%)		↓ (≈8.6 ± 5%)	Feraco et al., 2023 [148]
0.75 mM	16 h	BSA	1 µM × 10 min		↓ (≈6.3 ± 5%)		↓ (≈47.9 ± 5%)	Mazibuko-Mbeje et al., 2022 [149]
0.75 mM	16 h	BSA	100 nM × 10 min	↓ (≈60.6 ± 5%)	↓ (≈44.4 ± 5%)	↓ (≈80.0 ± 5%)		Chen et al., 2021 [150]
0.75 mM	16 h	BSA	100 nM × unknown				↓ (≈70.8 ± 5%)	Zhang et al., 2021 [151]
0.75 mM	16 h	BSA	No		↔ (≈70.0 ± 5%) SEM			Bitsi, 2020 [152]
0.75 mM	16 h	BSA	100 nM × 30 min		↓ (≈55.1 ± 5%) SEM			Bitsi, 2020 [152]
0.75 mM	16 h	BSA	100 nM × 15 min		↓ (≈47.8 ± 5%) SEM			de Mendonça et al., 2020 [153]
0.75 mM	16 h	BSA	No				↔ (≈112.1 ± 5%) SEM	Huang et al., 2019 [154]
0.75 mM	16 h	BSA	10 nM × 30 min		↓ Thr308 (≈57.5 ± 5%) SEM			Huang et al., 2019 [154]
0.75 mM	16 h	BSA	10 nM × 30 min		↓ Ser473 (≈44.1 ± 5%) SEM		↓ (≈95.3 ± 5%) SEM	Huang et al., 2019 [154]
0.75 mM	16 h	BSA	No			↓ (≈6.3 ± 5%)		Ayeleso et al., 2018 [155]
0.75 mM	16 h	BSA	100 nM × 30 min	↓ (≈59.0 ± 5%)	↔ (≈70.8 ± 5%)			Xu et al., 2017 [156]
0.75 mM	16 h	Unconjugated	100 nM × 30 min				↓ (≈92.0 ± 5%) SEM	Lee et al., 2017 [58]
0.75 mM	16 h	BSA	100 nM × 30 min				↓ (≈31.8 ± 5%) SEM	Lee et al., 2017 [58]
0.75 mM	16 h	BSA	Yes, unknown conditions	↓ (≈32.0 ± 5%)	↓ (≈36.0 ± 5%)		↓ (≈56.5 ± 5%)	Zhou et al., 2017 [157]
0.75 mM	16 h	BSA	100 nM × 30 min	↓ (≈26.3 ± 5%)	↓ Thr308 (≈54.0 ± 5%)	↓ (≈13.7 ± 5%)	↓ (≈71.4 ± 5%)	Zhu et al. [158]
0.75 mM	16 h	BSA	100 nM × 30 min		↓ Ser473 (≈64.8 ± 5%)			Zhu et al. [158]
0.75 mM	16 h	BSA	No				↔ (≈93.3 ± 5%)	Haghani et al., 2015 [159]
0.75 mM	16 h	BSA	100 nM × 30 min	↓ (≈58.9 ± 5%)	↓ (≈38.6 ± 5%)		↓ (≈62.7 ± 5%)	Haghani et al., 2015 [159]
0.75 mM	16 h	BSA	No				↔ (≈89.6 ± 5%)	Li et al. [160]
0.75 mM	16 h	BSA	100 nM × 30 min	↓ (≈40.4 ± 5%)	↓ (≈36.9 ± 5%)	↓ VC	↓ (≈70.2 ± 5%)	Li et al. [160]
0.75 mM	16 h	BSA	No		↓ (≈40.0 ± 5%)	↓ (≈42.0 ± 5%)	↓ (≈59.5 ± 5%)	Chen et al., 2015 [161]
0.75 mM	16 h	BSA	10 nM × 15 min		↓ (≈55.3 ± 5%)	↓ (≈43.4 ± 5%)	↓ (≈30.5 ± 5%)	Chen et al., 2015 [161]
0.75 mM	16 h	BSA	100 nM × 10 min		↓ (≈40.0 ± 5%)			Mahfouz et al., 2014 [162]
0.75 mM	16 h	BSA	No		↓ VC			Fabre et al., 2014 [163]
0.75 mM	16 h	BSA	1 nM × 10 min		↓ VC			Fabre et al., 2014 [163]
0.75 mM	16 h	BSA	No		↓ (≈33.3 ± 5%)	↓ (≈33.3 ± 5%)	↓ (≈33.3 ± 5%)	Mazibuko et al., 2013 [164]
0.75 mM	16 h	BSA	1 µM × 15 min		↓ (≈60.7 ± 5%)	↓ (≈15.9 ± 5%)	↓ (≈23.0 ± 5%)	Mazibuko et al., 2013 [164]
0.75 mM	16 h	BSA	No				↔ (≈93.7 ± 5%)	Zhang et al., 2010 [61]
0.75 mM	16 h	BSA	100 nM × 20 min				↓ (≈72.0 ± 5%)	Zhang et al., 2010 [61]
0.75 mM *	16 h	BSA	100 nM × 20 min	↑ (≈266.6 ± 5%)	↓ (≈43.5 ± 5%)	↑ (≈127.7 ± 5%)	↓ (≈73.7 ± 5%)	Zhang et al., 2010 [61]
0.75 mM	16 h	EtOH	No		↓ (≈50.0 ± 5%)			Peterson et al., 2008 [165]
0.75 mM	16 h	BSA	No		↓ VC			Chavez et al., 2003 [166]
0.75 mM	16 h	BSA	100 nM × 10 min		↓ VC			Chavez et al., 2003 [166]
0.75 mM	16 h	BSA	100 nM × 10 min		↓ VC			Chavez et al., 2005 [11]
0.75 mM *	16 h	BSA	100 nM × 10 min		↓ VC			Chavez et al., 2005 [11]
0.75 mM	16 h	BSA	100 nM × 10 min	↓ (≈20.0 ± 5%)	↓ VC			Jove et al., 2006 [167]
0.75 mM	16 h	BSA	No	↓ VC	↓ VC			Deng et al., 2012 [140]
0.75 mM	16 h	BSA	100 nM × 10 min	↓ VC	↓ VC		↓ (≈85.4 ± 5%)	Deng et al., 2012 [140]
0.75 mM	16 h	BSA	100 nM × 10 min		↓ VC			Hage Hassan et al., 2012 [60]
0.75 mM *	16 h	BSA	100 nM × 10 min		↓ (≈48. ± 5%)			Hage Hassan et al., 2012 [60]
0.75 mM	18 h	BSA	Unknown		↓ (≈77.1 ± 5%)			Aguer et al., 2014 [168]
0.75 mM *	18 h	BSA	10 nM × 10 min		↓ (≈11.1 ± 5%)			Wang et al., 2009 [142]
0.75 mM	18 h	BSA	10 nM × 10 min		↓ (≈32.1 ± 5%)			Wang et al., 2009 [142]
0.75 mM	18 h	BSA	No		↔ (≈100.0 ± 5%)	↔ (≈100.0 ± 5%)		Li et al., 2013 [169]
0.75 mM	18 h	BSA	100 nM × 30 min		↓ (≈16.6 ± 5%)	↓ (≈21.6 ± 5%)		Li et al., 2013 [169]
0.75 mM	19 h	BSA	10 nM × 10 min		↓ VC			Blackburn et al., 2020 [28]
0.75 mM	19 h	BSA	100 nM × 10 min		↓ VC			Blackburn et al., 2020 [28]
0.75 mM	24 h	BSA	100 nM × 15 min		↓ VC	↓ VC	↓ (≈60.6 ± 5%)	Chen et al., 2016 [170]
0.75 mM	24 h	BSA	100 nM × 30 min	↑ (≈164.1 ± 5%)		↓ (≈50.7 ± 5%)		Chen et al., 2020 [171]
0.75 mM	24 h	BSA	No			↓ (≈63.1 ± 5%)		Zhang et al., 2024 [83]
0.75 mM	24 h	BSA	100 nM × 30 min				↓ (≈47.3 ± 5%)	Zhang et al., 2024 [83]
0.75 mM	24 h	BSA	100 nM × 10 min				↓ (≈70.0 ± 5%)	Swargiary et al., 2024 [172]
0.75 mM	24 h	BSA	10 nM × 10 min		↓ (≈56.5 ± 5%)			De Hart et al., 2023 [141]
0.75 mM	24 h	BSA	No				↔ (≈89.4 ± 5%)	Zhang et al., 2021 [173]
0.75 mM	24 h	BSA	30 mU/mL × 60 min	↓ (≈18.1 ± 5%)	↓ (≈17.6 ± 5%)		↓ (≈66.6 ± 5%)	Zhang et al., 2021 [173]
0.75 mM	24 h	BSA	No			↓ (≈53.1 ± 5%)	↓ (≈67.2 ± 5%)	Wang et al., 2021 [174]
0.75 mM	24 h	BSA	100 nM × 30 min			↓ (≈52.0 ± 5%)	↓ (≈61.1 ± 5%)	Wang et al., 2021 [174]
0.75 mM	24 h	BSA	No	↓ VC	↓ VC			Han et al., 2020 [175]
0.75 mM	24 h	BSA	Yes, unknown	↓ VC	↓ VC			Han et al., 2020 [175]
0.75 mM	24 h	BSA	100 nM × unknown				↓ (≈14.0 ± 5%)	Chen et al., 2019 [129]
0.75 mM	24 h	BSA	No	↓ (≈40.9 ± 5%)		↓ (≈51.7 ± 5%)		Zhang et al., 2018 [17]
0.75 mM	24 h	BSA	No				↓ (≈28.5 ± 5%)	Bakar et al., 2017 [176]
0.75 mM	24 h	BSA	100 nM × 30 min		↓ (≈38.2 ± 5%)		↓ (≈20.0 ± 5%)	Bakar et al., 2017 [176]
0.75 mM	24 h	BSA	No	↑ VC	↓ VC		↓ (≈64.8 ± 5%)	Kwak et al., 2017 [177]
0.75 mM	24 h	BSA	1 μg/mL × 30 min	↑ VC	↓ VC		↓ (≈57.1 ± 5%)	Kwak et al., 2017 [177]
0.75 mM	24 h	BSA	10 nM × 30 min		↓ (≈25.6 ± 5%)			Huang et al., 2017 [133]
0.75 mM	24 h	BSA	No	↔ (≈236.0 ± 5%)	↔ (≈50.0 ± 5%)		↔ (≈47.8 ± 5%)	Li et al., 2017 [178]
0.75 mM	24 h	BSA	100 nM × 30 min	↓ (≈256.0 ± 5%)	↓ (≈45.7 ± 5%)		↓ (≈40.4 ± 5%)	Li et al., 2017 [178]
0.75 mM	24 h	BSA	No	↓ (≈21.4 ± 5%)	↓ (≈11.1 ± 5%)	↔ (≈92.3 ± 5%)	↔ (≈78.5 ± 5%)	Kwak et al., 2016 [179]
0.75 mM	24 h	BSA	1 μg/mL × 30 min	↓ (≈21.5 ± 5%)	↓ (≈39.2 ± 5%)	↓ (≈50.0 ± 5%)	↓ (≈61.3 ± 5%)	Kwak et al., 2016 [179]
0.75 mM	24 h	BSA	No				↔ (≈90.0 ± 5%)	Meshkani et al., 2014 [180]
0.75 mM	24 h	BSA	100 nM × 30 min		↓ (≈62.9 ± 5%)		↓ (≈70.5 ± 5%)	Meshkani et al., 2014 [180]
0.75 mM	24 h	BSA	No		↓ VC			Dymkowska et al., 2014 [181]
0.75 mM	24 h	BSA	10 nM × 20 min		↓ (≈51.7 ± 5%)		↓ (≈83.0 ± 5%)	Dymkowska et al., 2014 [181]
0.75 mM	24 h	BSA	10 nM × 10 min		↓ (≈9.7 ± 5%)			Wang et al., 2009 [142]
0.75 mM	24 h	BSA	10 nM × 10 min		↓ (≈16.3 ± 5%)			Senn et al., 2006 [182]
0.75 mM	48 h	BSA	No		↓ (≈39.1 ± 5%)			Yu et al., 2024 [183]
0.8 mM	18 h	BSA	No		↔ (≈90.9 ± 5%)			D’Souza et al., 2018 [184]
0.8 mM	18 h	BSA	20 nM × 15 min		↓ (≈70.2 ± 5%)			D’Souza et al., 2018 [184]
0.8 mM	18 h	BSA	200 nM × 30 min	↑ (≈271.4 ± 5%)	↑ (≈131.8 ± 5%)			Lui et al., 2012 [185]
1 mM	16 h	BSA	No		↓ VC		↑ (≈207.4 ± 5%) SEM	Kadotani et al., 2008 [186]
1 mM	16 h	BSA	2 nM × 30 min		↓ VC			Kadotani et al., 2008 [186]
1 mM	16 h	BSA	10 nM × 30 min		↓ VC			Kadotani et al., 2008 [186]
1 mM	16 h	BSA	100 nM × 30 min		↓ VC		↓ (≈78.2 ± 5%) SEM	Kadotani et al., 2008 [186]
1 mM	16 h	Unconjugated	100 nM × 30 min				↓ (≈84.0 ± 5%) SEM	Lee et al., 2017 [58]
1 mM	16 h	BSA	100 nM × 30 min				↓ (≈30.6 ± 5%) SEM	Lee et al., 2017 [58]
1 mM	16 h	BSA	No		↓ (≈76.1 ± 5%)			Zhou et al., 2007 [59]
1 mM	16 h	BSA	No	↑ (≈191.8 ± 5%)	↔ (≈150.0 ± 5%)		↓ (≈75.8 ± 5%)	Ragheb et al., 2009 [187]
1 mM	16 h	BSA	100 nM × 10 min	↑ (≈159.0 ± 5%)	↓ (≈72.2 ± 5%)		↓ (≈60.0 ± 5%)	Ragheb et al., 2009 [187]
1 mM	16 h	BSA	100 nM × 30 min		↓ VC			Tsuchiya et al., 2010 [188]
1 mM	16 h	BSA	100 nM × 10 min		↓ (≈42.8 ± 5%)			Kusudo et al., 2011 [189]
1 mM	16 h	BSA	No				↔ (≈90.6 ± 5%)	Zhang et al., 2010 [61]
1 mM	16 h	BSA	100 nM × 20 min				↓ (≈72.0 ± 5%)	Zhang et al., 2010 [61]
1 mM	17 h	BSA	100 nM × 10 min		↓ Thr308 (≈16.6 ± 5%)			Pierre et al., 2016 [190]
1 mM	17 h	BSA	100 nM × 10 min		↓ Ser473 (≈29.6 ± 5%)			Pierre et al., 2016 [190]
1 mM	24 h	BSA	No			↔ (≈103.2 ± 5%)	↔ (≈119.2 ± 5%)	Vong et al., 2025 [191]
1 mM	24 h	BSA	500 nM × unknown			↓ (≈80.8 ± 5%)	↓ (≈81.6 ± 5%)	Vong et al., 2025 [191]
1 mM	24 h	BSA	100 nM × 30 min				↓ (≈37.9 ± 5%)	Kim et al., 2024 [192]
1 mM	24 h	BSA	No			↓ (≈24.5 ± 5%)	↓ (≈40.0 ± 5%)	Xiang et al., 2023 [193]
1 mM	24 h	BSA	100 nM × 30 min			↓ Cytosolic (≈78.1 ± 5%)		Kim et al., 2020 [194]
1 mM	24 h	BSA	100 nM × 30 min	↑ (≈147.2 ± 5%)	↓ (≈36.6 ± 5%)	↓ Membranous (≈42.4 ± 5%)	↓ (≈53.2 ± 5%)	Sun et al., 2020 [195]
1 mM	48 h	BSA	17 nM × 15 min	↓ (≈24.5 ± 5%)	↓ (≈20.8 ± 5%)			Zhou et al., 2008 [196]
1 mM	48 h	BSA	100 nM × 15 min	↓ (≈40.7 ± 5%)	↓ (≈68.6 ± 5%)	↓ (≈42.6 ± 5%)		Sun et al., 2020 [195]
1.2 mM	16 h	BSA	100 nM × 15 min	↓ (≈72.3 ± 5%) SEM	↓ (≈55.8 ± 5%) SEM			Hsieh et al., 2014 [145]

NOTES: * indicates aspects of results were repeated with separate experiment within the same report using similar treatment conditions. NR indicates detail was not reported. VC indicates visual confirmation was used and statistical comparisons were not made for relevant groups or only used single image densitometry. Abbreviations: bovine serum albumin (BSA). ↓: indicates a reported decrease in the target versus control; ↔: indicates no difference reported in the target versus control; ↑: indicates a reported increase in the target versus control.

## Data Availability

No new data were created or analyzed in this study.

## Supplementary Materials

The following supporting information can be downloaded at: https://www.mdpi.com/article/10.3390/nu17223619/s1, Table S1: Composite search results and inclusion/exclusion results from Pubmed and Scopus.; File S1: Raw data estimates of original research [4,7,8,9,10,11,12,13,14,15,16,17,18,19,20,21,22,23,24,25,26,27,28,29,30,31,32,33,34,35,36,37,38,39,40,41,42,43,44,45,46,47,48,49,50,51,52,53,54,55,56,57,58,59,60,61,62,63,64,65,66,67,68,69,70,71,72,73,74,75,76,77,78,79,80,81,82,83,84,85,86,87,88,89,90,91,92,93,94,95,96,97,98,99,100,101,102,103,104,105,106,107,108,109,110,111,112,113,114,115,116,117,118,119,120,121,122,123,124,125,126,127,128,129,130,131,132,133,134,135,136,137,138,139,140,141,142,143,144,145,146,147,148,149,150,151,152,153,154,155,156,157,158,159,160,161,162,163,164,165,166,167,168,169,170,171,172,173,174,175,176,177,178,179,180,181,182,183,184,185,186,187,188,189,190,191,192,193,194,195,196].

## Abbreviations

The following abbreviations are used in this manuscript:

3D	3 dimensional
Glut4	glucose transporter member 4
GSV	glut4 storage vesicles
HG	high glucose
Iκκ	I kappa B kinase
IRS1	insulin receptor substrate 1 (protein)
IR	insulin receptor (protein)
LG	low glucose
PI3K	phosphatidylinositol 3-Kinase (protein)
PAkt	phosphorylated Akt
PKC	protein kinase c
ROS	reactive oxygen species
SD	standard deviation
SEM	standard error of the mean
TLR4	toll-like receptor 4
VC	visual confirmation
