# Supplementary material for: A Systematic Review of Palmitate-Mediated Insulin Resistance in C2C12 Myotubes
Source: Nutrients. 2025 Nov 20;17(22):3619. doi: 10.3390/nu17223619 (PMC12655210; doi:10.3390/nu17223619)
Supplement: Supplementary file 1 [file nutrients-17-03619-s001.zip › Zimmerman Nutrients - Table s1 10-22-2025 R1.pdf]

Table S1. Composite search results and inclusion/exclusion results from Pubmed and Scopus.

	**Search Terms**			**Inclusion/Exclusion Results**								**Total Search Result**
	**Primary Search Terms**	**Secondary Search Terms**	**Tertiary Search Terms**	**Outcomes / Treatment**	**Language**	**Unavailable**	**Retraction**	**Abstract**	**Review**	**Redundant**	**Included**	
**PUBMED Search Results**	**C2C12 AND Palmitate**	IR		51	3	6	2		1	0	161	224
	**C2C12 AND Palmitate**	IR	pAkt							11		11
	**C2C12 AND Palmitate**	IR	IRS1							25		25
	**C2C12 AND Palmitate**	IR	GLUT4							42		42
	**C2C12 AND Palmitate**		pAkt	1						14		15
	**C2C12 AND Palmitate**		IRS							26		26
	**C2C12 AND Palmitate**		GLUT4	3		1				45	1	50
	**C2C12 AND Palmitic Acid**	IR		1						94	1	96
	**C2C12 AND Palmitic Acid**	IR	pAkt							8		8
	**C2C12 AND Palmitic Acid**	IR	IRS1							14		14
	**C2C12 AND Palmitic Acid**	IR	GLUT4							24		24
	**C2C12 AND Palmitic Acid**		pAkt							9		9
	**C2C12 AND Palmitic Acid**		IRS							14		14
	**C2C12 AND Palmitic Acid**		GLUT4							28		28
	**PUBMED Totals**			**56**	**3**	**7**	**2**	**0**	**1**	**354**	**163**	**586**
**SCOPUS Search Results**	**C2C12 AND Palmitate**	IR		6	9		2	1		172	6	196
	**C2C12 AND Palmitate**	IR	pAkt							2	1	3
	**C2C12 AND Palmitate**	IR	IRS1		1					23		24
	**C2C12 AND Palmitate**	IR	GLUT4		1					32	2	35
	**C2C12 AND Palmitate**		pAkt							3		3
	**C2C12 AND Palmitate**		IRS							25		25
	**C2C12 AND Palmitate**		GLUT4							39		39
	**C2C12 AND Palmitic Acid**	IR		10	2					223	11	246
	**C2C12 AND Palmitic Acid**	IR	pAkt							3		3
	**C2C12 AND Palmitic Acid**	IR	IRS1	1	3					23	3	30
	**C2C12 AND Palmitic Acid**	IR	GLUT4	1	1					43	1	46
	**C2C12 AND Palmitic Acid**		pAkt							3		3
	**C2C12 AND Palmitic Acid**		IRS							30	2	32
	**C2C12 AND Palmitic Acid**		GLUT4	7						45	2	54
	**SCOPUS Totals**			**25**	**17**	**0**	**2**	**1**	**0**	**666**	**28**	**739**
	**All Search Totals**			**81**	**20**	**7**	**4**	**1**	**1**	**1020**	**191**	**1325**
